# Optimal Effective Concentration Combinations and synergy evaluations for binary antimicrobial combinations *in vitro*

**DOI:** 10.3389/fmicb.2025.1645341

**Published:** 2025-10-01

**Authors:** Lars Michael Greger, Christoph Greger, Karl-Anton Hiller, Tim Maisch

**Affiliations:** ^1^Department of Conservative Dentistry and Periodontology, University Hospital Regensburg, Regensburg, Germany; ^2^Department of Dermatology, University Hospital Regensburg, Regensburg, Germany; ^3^Faculty of Informatics and Data Science, University of Regensburg, Regensburg, Germany

**Keywords:** Optimal Effective Concentration Combination (OPECC), Bliss independence, Loewe additivity, synergy, effective antimicrobial combination, bacterial resistance, checkerboard assay

## Abstract

**Background:**

This study provides a comparative analysis of Optimal Effective Concentration Combinations (OPECCs) and synergy evaluations derived from the Loewe additivity and Bliss independence models for binary antimicrobial combinations *in vitro*. The aim was to provide a comprehensive perspective on the utility of these strategies in analyzing binary antimicrobial combinations and their implications for effective therapeutic strategies. This study contributes to the understanding of methodological differences in evaluating antimicrobial combinations.

**Methods:**

Binary combinations of Benzalkonium chloride, Chlorhexidine, Cetylpyridinium chloride, and Ciprofloxacin were tested against *E. coli* and *S. aureus*. OPECCs and synergy evaluations were derived from OD-measurements after 3 h of aerobic incubation at 37 °C in Mueller-Hinton medium.

**Results:**

All OPECCs were determinable for each binary combination pair. For each binary concentration component, the OPECC lay below the respective minimum effective concentration in single application. The synergy scores obtained with both models ranged from −13.4 (antagonistic) to 11.2 (synergistic), with consistently higher scores for the Bliss model. However, the concentration pairs at maximum synergy, determined using the respective matrices, showed inconsistent antibacterial assessments. No pattern could be derived regarding the antibacterial effect of these concentrations in relation to the OPECCs, nor between the two synergy models. The general synergy score of a combination also does not inevitably reflect the results at effective concentrations.

**Conclusion:**

The comparison demonstrated that the assumptions like “additivity” or “independence” underlying these models can result in concentration pairs at maximum synergy that may not necessarily be effective. As a consequence, the synergy evaluation methods tested do not account for the effectiveness of the assessed concentration pairs. In contrast, the model-independent OPECC method identifies effective concentration combinations directly from experimental data, without reliance on interaction assumptions or further data processing. The separating curve is based on directly measured optical density (OD) values of the binary concentration combinations, thus representing the real situation. By offering an alternative or complementary approach to existing models, the OPECC method may support more accurate identification of effective antimicrobial combinations and provide valuable insights for the development of optimized treatment strategies in the context of rising antimicrobial resistance.

## 1 Introduction

Antimicrobial resistance (AMR) poses a significant global threat to public health (Geneva: World Health Organization, 2022; Laxminarayan, 2022; Tacconelli and Pezzani, 2019), resulting from excessive and improper use of antimicrobial substances across animal, plant, and human domains (Holmes et al., 2016; Olesen et al., 2018). In 2021 alone, estimations suggested that bacterial AMR was linked to approximately 4.71 million deaths, with 1.14 million deaths being directly caused by AMR (GBD 2021 Antimicrobial Resistance Collaborators, 2024). The 2022 Global Antimicrobial Resistance and Use Surveillance System (GLASS) report underlines the alarming level of resistant strains among prevalent bacterial pathogens. Notably, in 76 countries, median reported resistance rates reached 42% for third-generation cephalosporin-resistant *Escherichia coli* and 35% for methicillin-resistant *Staphylococcus aureus*, posing significant concerns (Geneva: World Health Organization, 2022). Projections suggest that by 2050, AMR could directly result in about 1.91 million deaths and be associated with as many as 8.22 million deaths worldwide (GBD 2021 Antimicrobial Resistance Collaborators, 2024).

The repercussions of antimicrobial resistance extend beyond health, including profound economic implications. The World Bank projects that by 2050, AMR could lead to an additional US$ 1 trillion in healthcare costs and annual gross domestic product losses ranging from US$ 1 trillion to US$ 3.4 trillion by 2030 (Jonas et al., 2017). Moreover, this problem is exacerbated by the lack of new antimicrobial agents (Coates et al., 2020; Prestinaci et al., 2015). To mitigate the rise of resistance in human health, various strategies can be employed. Ensuring optimal drug concentrations and repurposing underutilized drugs are two methods proposed to delay the development of tolerance or resistance (Holmes et al., 2016). Maintaining diversity in antimicrobial use may also be a method to prevent selective pressure, which can be achieved by implementing heterogeneous approaches such as drug cycling within hospitals or units, as well as individual-level drug mixing (Coates et al., 2020; Holmes et al., 2016). Combinations of different antimicrobial substances (AMS) can exhibit effects that differ from what would be expected through the single application of the AMS (Choudhary et al., 2022; Coates et al., 2020; Keith et al., 2005). To evaluate drug combinations, various mathematical models can be used as so-called null reference models, which predict a reaction that is then compared with experimentally obtained data. These models are often defined based on the knowledge of the different possibilities of interactions between AMS and their targets or between themselves (Berenbaum, 1989; Bliss, 1939; Chou, 2006; Greco et al., 1995; Loewe and Muischnek, 1926; Yadav et al., 2015). Both the Loewe additivity and Bliss independence model are frequently recommended as null reference models to evaluate the effects of drug combinations (Greco et al., 1995; Yadav et al., 2015), yielding differing results depending on the interaction mechanism of the AMS with each other and the microbials (Baeder et al., 2015; Goldoni and Johansson, 2007). For Bliss independence, the assumption made is the “probabilistic independence” (Greco et al., 1995) of the mechanisms of action of the AMS. This assumption means that if two drugs act independently, their combined effect, measured in terms of remaining viability or survival fraction, should be equal to the product of their individual effects (Bliss, 1939, 1956). Loewe additivity, on the other hand, assumes that a drug cannot interact with itself. The model is based on a sham experiment, in which a drug is hypothetically combined with itself to establish a baseline for non-interaction. According to this principle, when two drugs with the same effect are used together, their combined effect should be their expected additive effect (Greco et al., 1995; Loewe and Muischnek, 1926; Tang et al., 2015). There are various web-applications and software available, such as *SynergyFinder* (Ianevski et al., 2022) and *Combenefit* (Di Veroli et al., 2016), that can be used to apply these models. A comparison between observed growth or inhibition and the predicted outcomes, using measures such as the “lack-of-fit” (Lederer et al., 2019), is used to quantify synergy.

The tested concentration pairs can be analyzed to determine the observed maximum synergy (e.g., *Combenefit*: *SYN_MAX metric*) (Di Veroli et al., 2016; Khandelwal Gilman et al., 2021). This includes identifying the corresponding concentration pair at maximum synergy. Additionally, the overall effect of the drug combination can be evaluated using a synergy score [e.g., *Combenefit*: *SUM_SYN_ANT* or *SUM_SYN_ANT_WEIGHTED metric* (Di Veroli et al., 2016)], which provides a synergy assessment independent of the specific tested concentrations (Khandelwal Gilman et al., 2021). According to Khandelwal Gilman et al. (2021), a *SUM_SYN_ANT metric* score greater than 2.0 indicates synergy, a score between −2.0 to 2.0 is considered neutral [additivity for Loewe, independence for Bliss (Tang et al., 2015)], and a score below −2.0 suggests antagonism (Khandelwal Gilman et al., 2021).

To identify interactions between two agents, the checkerboard assay (broth microdilution test) can be used, which involves serially diluting two antimicrobial agents in a two-dimensional manner to cover multiple combinations within relevant ranges (Jenkins and Schuetz, 2012; Wozniak and Grinholc, 2018). Antimicrobial substances have specific targets within bacteria, aiming to hinder their growth or to eliminate them altogether. These targets often include essential processes like DNA or RNA synthesis, cell wall construction, and protein synthesis. They may also target the integrity of the outer membrane or interfere with central cell metabolism (Choudhary et al., 2022). Benzalkonium chloride (BAC), Chlorhexidine (CHX), and Cetylpyridinium chloride (CPC) are commonly used as detergents in clinical environments (Cieplik et al., 2019; Le Gilbert, 2005; LeBel et al., 2020; Mao et al., 2020). These substances exhibit a similar mechanism of action by interacting with the negatively charged bacterial membrane, which is stabilized by various cations such as Ca^2+^ and Mg^2+^. The bacterial membrane carries this inherent negative charge due to components such as lipoteichoic acid in Gram-positive bacteria or lipopolysaccharides in Gram-negative bacteria, as well as the phospholipids of the lipid bilayer membrane itself. This negative charge provides a potential site of interaction for quaternary ammonium compounds such as CPC, BAC, and the bis-biguanide CHX, which initially displace these ions with their positively charged counterparts. Consequently, this results in membrane destabilization, leakage, and subsequent bactericidal effects when the agent concentration is sufficient (Hiller et al., 2023; Le Gilbert, 2005). CHX, with two positive charges, demonstrates stronger binding to bacterial surfaces compared to BAC and CPC, each carrying a single positive charge. The hydrophobic regions of quaternary ammonium compounds integrate into the bacterial membrane. This differs from CHX, which primarily acts on the outer surface of the membrane rather than integrating itself into it (Cieplik et al., 2019; Le Gilbert, 2005; Muehler et al., 2017). Ciprofloxacin (CIP) is a fluoroquinolone known for its bactericidal properties and its ability to inhibit nucleic acid synthesis. It is commonly used to treat infections caused by a wide range of aerobic Gram-positive and aerobic Gram-negative microorganisms, including *E. coli* and *S. aureus* (Ball, 1986; Zhang et al., 2018). The drugs antimicrobial action stems from its ability to inhibit DNA gyrase (a type of topoisomerase II) in *E. coli.* It primarily targets topoisomerase IV (also a topoisomerase II) in *S. aureus*, thereby interfering with bacterial DNA synthesis. Inhibition of topoisomerase IV decreases the rate of replication elongation and results in a slower inhibition of growth than inhibition of DNA gyrase (Dodd-Butera and Broderick, 2014; Ferrero et al., 1994; Hirsch and Klostermeier, 2021; NG et al., 1996).

A method to determine an *Optimal Effective Concentration Combination* (OPECC) for binary antimicrobial combinations was introduced by our group (Hiller et al., 2023). This approach does not require any assumptions such as additivity or independence of the substances’ modes of action. Additionally, the method enables the distinction between effective and not effective concentration pairs. However, OPECC does not incorporate a synergy evaluation (Hiller et al., 2023).

To determine the OPECC, binary antimicrobial concentration data from a checkerboard assay are plotted as a three-dimensional surface, with optical density (normalized OD) as a function of the two drug concentrations. The contour line representing the boundary between effectiveness and bacterial growth is extracted and projected into the concentration plane with 90% confidence intervals. Along this line of effective concentration pairs, a decrease in one agent’s concentration requires an increase in the other to maintain efficacy. The most balanced trade-off in concentration terms without placing the two AMS into a direct quantitative relationship occurs at the curve’s inflection point, which defines the OPECC. When such an inflection point is detectable, this method identifies the combination that achieves eradication with both agents maximizing efficiency without assuming a specific interaction mechanism. All other points on this curve or to the top right thereof are effective, but not optimal. All concentration pairs to the bottom left of this curve are not effective (Hiller et al., 2023).

This study aimed to identify *Optimal Effective Concentration Combinations* (OPECCs) of binary antimicrobial combinations *in vitro* and to compare these results with synergy scores and concentration pairs at maximum synergy, as determined with the Loewe additivity and Bliss independence models. The findings show that the conditions of “additivity” (Loewe and Muischnek, 1926) or “independence” (Bliss, 1939) required by these models can lead to concentration pairs at maximum synergy that may not necessarily be effective. As a consequence, the synergy evaluation methods tested do not account for the effectiveness of the assessed concentration pairs. Furthermore, the general synergy score of a combination does not inevitably reflect the results at effective concentrations. In contrast, the OPECC calculation, a model-independent method that does not require specific knowlegde about the mechanisms of action of the substances, can distinguish between effective and not effective combinations and determine the optimal effective concentration in each situation without the need to include a synergy assessment.

## 2 Materials and methods

### 2.1 *E. coli* and *S. aureus* strains and growth conditions

*Escherichia coli* (ATCC 25922) and *Staphylococcus aureus* (ATCC 25923) were procured from the American Type Culture Collection (Manassas, VA, USA). The bacteria strains were grown and stored on Müller-Hinton agar plates (MH; provided by the Institute of Clinical Microbiology and Hygiene, University Hospital Regensburg, Germany; consisting of 38.0 g of powdered Müller-Hinton agar, ready to use (Merck KGaA, Darmstadt, Germany; composed of agar (17.0 g/L), beef infusion substances (2.0 g/L), casein hydrolysate (17.5 g/L), and starch (1.5 g/L)), dissolved in 1 L of Millipore water). A single colony of bacteria was selected from the agar plate and transferred to 5 mL of medium for an overnight (o/n) culture on an orbital shaker at 37 °C (180 rpm). Subsequently, the o/n planktonic cultures were centrifuged at 1200 rpm for 5 min, the supernatant was removed, and the pellets were dissolved in ion phosphate-buffered saline (PBS; Dulbecco’s Phosphate Buffered Saline; Sigma-Aldrich, St. Louis, MO, USA). The bacterial suspension was adjusted to an optical density (OD) of 0.6, measured at 600 nm (SPECORD 50 Plus, Analytik Jena, Jena, Germany) (Hiller et al., 2023).

### 2.2 Antibacterial compounds

The antibacterial compounds Benzalkonium chloride (BAC), Chlorhexidine (CHX) and Cetylpyridinium chloride (CPC) were purchased from Sigma Aldrich (Merck KGaA, Darmstadt, Germany). Stock solutions of the antimicrobials BAC and CPC were prepared in *aqua dest*. and adjusted to the concentration of 128 μg/mL. For CHX, a stock solution of 2% (w-v) (20.000 μg/mL) was used (Hiller et al., 2023). Ciprofloxacin (CIP) was procured from Fresenius KABI (Bad Homburg, Germany) as a 200 mg/100 mL infusion solution and was subsequently adjusted to a concentration of 200 μg/mL in *aqua dest.* with a pH of 4.8 (adjusted with HCl). All compounds were filter-sterilized (Rotilabo® Syringe Filters, Carl Roth GmbH & Co. KG, Germany) to a pore size of 0.2 μm. Serial dilutions in distilled water were prepared to determine sublethal and inhibitory concentrations for the subsequent combination experiments. For CIP, distilled water with a pH of 4.8 was consistently used.

### 2.3 Combination experiments

The combination experiments were conducted using a checkerboard assay on 48-well plates (Botelho, 2000; Wozniak and Grinholc, 2018). All six different combinations of BAC, CPC, CHX and CIP were tested for both bacterial strains. The dilution series were designed based on the sublethal and inhibitory concentrations of the individual compounds. These were determined in range-finding experiments using the classical empirical method and the “Area under the Curve Method” (Hiller et al., 2023). First, 300 μL of Mueller-Hinton (MH) broth was pipetted into each well. Then, 150 μL of each compound was added to achieve the desired final concentrations. For wells with a concentration of 0, the corresponding volume of distilled water, as used for dilution, was added. Finally, 50 μL of a bacterial suspension adjusted to an optical density (OD) of 0.6 was added. Four wells containing 650 μL of MH broth served as controls, along with an additional plate without bacteria for each possible combination. Optical density was determined spectroscopically (VarioSkan Flash, SkanIt v. 2.4.5, Thermo Fisher Scientific, Vantaa, Finland) at 600 nm both before and after incubation for 180 min. The plates were incubated at a temperature of 37 °C under aerobic conditions, without agitation, consistent with protocols previously established by our research group (Hiller et al., 2023). Three independent experiments were performed on the six respective combination pairs for each bacteria strain and 6 for CHX and CIP against *S. aureus*.

### 2.4 Evaluation of binary combinations

To evaluate the various combinations for synergy or antagonism, the Bliss independence and Loewe additivity models were applied using the software *Combenefit* (Di Veroli et al., 2016). OD_kor_, which was obtained by subtracting the OD values taken at minute zero from those at minute 180 was normalized to the 0 to 100 scale for growth (OD_proc_) at each combination point as required by *Combenefit*.

The results calculated with *Combenefit* were used to determine the concentration pairs at maximum synergy [*SYN_MAX metric* (Di Veroli et al., 2016)] for Bliss independence and Loewe additivity for the six combinations against the two strains each. In addition, the *SUM_SYN_ANT_WEIGHTED metric* (Di Veroli et al., 2016), further referenced as synergy score, was used to assess whether the combination considered as a whole exhibited synergism (positive values) or antagonism (negative values) for each model.

The *Optimal Effective Concentration Combinations* (OPECCs) were calculated using the OD_kor_ values, as previously established by our research group (Hiller et al., 2023). Briefly, the function and corresponding 90% confidence intervals in the concentration plane that separates the effective from the not effective concentrations were calculated (TableCurve 2D) and used to determine the OPECC, which is the inflection point. This curve was additionally used to classify the concentration pairs at maximum synergy for both models regarding their effectiveness.

## 3 Results

In this study, it was possible to distinguish between effective and not effective combinations and to identify the *Optimal Effective Concentration Combination* (OPECC) for each binary combination of compounds tested against both bacterial strains. For OPECC, each individual concentration component was below the minimum effective concentration of the given compound in single application (Table 1).

**Table 1:** The OPECC for all binary combinations of BAC, CHX, CPC, and CIP against *E. coli* and *S. aureus.*

Organism	Method	Compound 1 [μg/ml]		Compound 2 [μg/ml]	
*E. coli*	**OPECC**	BAC	**2.5 (2.5; 2.5)**	CHX	**0.7 (0.5; 0.8)**
	*Single* ^*^	BAC	*2.6 (2.6; 2.7)*	CHX	*1.4 (1.3; 1.4)*
	**OPECC**	BAC	**3.6 (3.5; 3.6)**	CIP	**0.038 (0.035; 0.041)**
	*Single*	BAC	*4.9 (4.7; 5.2)*	CIP	*0.081 (0.079; 0.083)*
	**OPECC**	BAC	**3.3 (3.2; 3.4)**	CPC	**1.8 (1.6; 2.0)**
	*Single*	BAC	*4.9 (4.3; −9^†^)*	*CPC*	*3.6 (3.5; 3.8)*
	**OPECC**	CHX	**1.3 (1.3; 1.3)**	CIP	**0.041 (0.038; 0.044)**
	*Single*	CHX	*1.6 (1.6; 1.6)*	CIP	*0.100 (0.102; 0.103)*
	**OPECC**	CPC	**3.0 (3.0; 3.1)**	CHX	**0.7 (0.6; 0.8)**
	*Single*	CPC	*3.8 (3.6; −9)*	CHX	*1.4 (1.4; 1.5)*
	**OPECC**	CPC	**3.9 (3.9; 4.0)**	CIP	**0.060 (0.058; 0.063)**
	*Single*	CPC	*6.1 (6.1; 7.0)*	CIP	*0.123 (0.121; 0.125)*
*S. aureus*	**OPECC**	BAC	**2.2 (2.2; 2.2)**	CHX	**0.6 (0.5; 0.6)**
	*Single*	BAC	*2.8 (2.4; −9)*	CHX	*1.1 (1.1; 1.2)*
	**OPECC**	BAC	**2.3 (2.3; 2.3)**	CIP	**17.5 (16.8; 18.2)**
	*Single*	BAC	*2.8 (2.7; −9)*	CIP	*35.2 (32.1; 38.3)*
	**OPECC**	BAC	**2.2 (2.2; 2.2)**	CPC	**0.5 (0.5; 0.6)**
	*Single*	BAC	*3.3 (2.9; −9)*	CPC	*0.9 (0.9; 1.0)*
	**OPECC**	CHX	**1.3 (1.3; 1.3)**	CIP	**15.1 (13.7; 16.5)**
	*Single*	CHX	*1.6 (1.5; −9)*	CIP	*30.4 (27.7; 33.1)*
	**OPECC**	CPC	**0.9 (0.9; 0.9)**	CHX	**0.7 (0.6; 0.8)**
	*Single*	CPC	*1.1 (1.0; −9)*	CHX	*1.3 (1.3; 1.4)*
	**OPECC**	CPC	**1.0 (1.0; 1.0)**	CIP	**16.4 (15.8; 16.9)**
	*Single*	CPC	*1.3 (1.2; −9)*	CIP	*32.7 (31.1; 34.3)*

The table presents the calculated OPECC (bold) using the protocols previously established by our group (Hiller et al., 2023) for all tested binary combinations of BAC, CHX, CPC, and CIP against *E. coli* and *S. aureus*, as well as the minimum effective concentration of the compound in single application (*italic*). The results include 90% confidence intervals for each binary combination in parentheses.

*Single, minimum effective concentration of the given compound in single application.

†-9, Value represents undeterminable results (the respective curve for the confidence interval did not intersect the abscissa).

**Figure 1:**
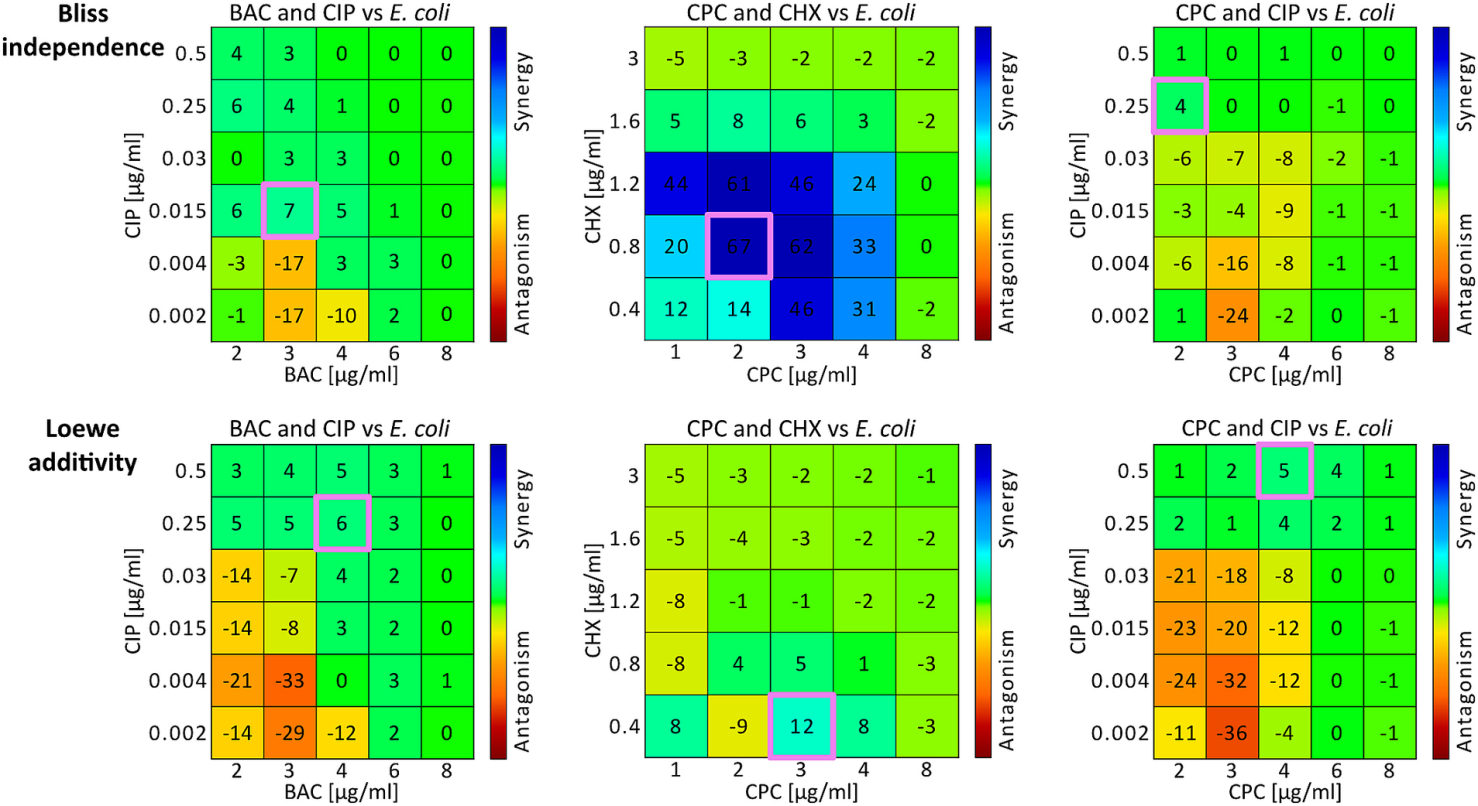
Synergy matrices and concentration pairs at maximum synergy from *E. coli* experiments. Three examples are illustrated using the combinations BAC and CIP (left column), CPC and CHX (middle column), and CPC and CIP (right column) against *E. coli*. The two rows present the *Combenefit* synergy matrices for Bliss independence (top row) and Loewe additivity (bottom row), displaying drug combinations with marked areas indicating specific interaction values. The color gradient represents synergy (blue) to antagonism (red). The concentration pairs at maximum synergy for both models are each highlighted in pink (Table 2). For Bac and CIP (left column) the synergy scores were 0.5 for Bliss independence and −2.2 for Loewe additivity. For CPC and CHX (middle column) the synergy scores were −3.1 for Bliss independence and −6.8 for Loewe additivity. For CPC and CIP (right column) the synergy scores were 9.4 for Bliss independence and −0.2 for Loewe additivity (Table 2).

**Figure 2:**
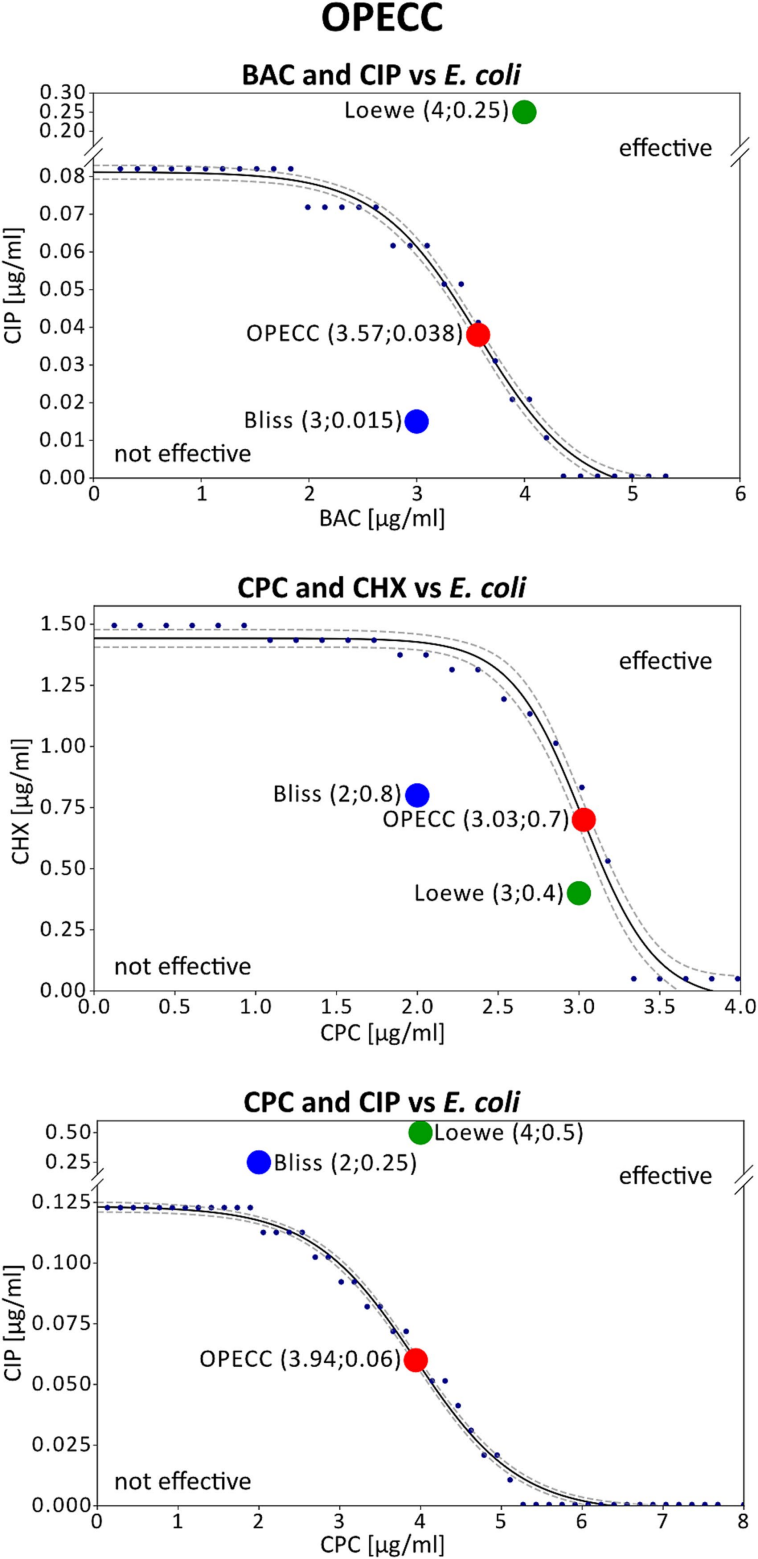
Concentration pairs at maximum synergy and OPECCs from *E. coli* experiments. The three potential outcomes are illustrated using the combinations BAC and CIP (top), CPC and CHX (middle), and CPC and CIP (bottom) against *E. coli*. Displayed is the curve separating the effective from the not effective concentrations (solid line), that was used to calculate the OPECC (red dot: inflection point), along with the corresponding concentration pairs at maximum synergy based on Bliss independency (blue dot) and Loewe additivity (green dot). The same combinations of compounds were depicted in Figure 1 and Figure 2. The concentration pairs at maximum synergy used here are highlighted pink in Figure 1. The OPECC curve (solid line) was fitted using a four-parameter sigmoid model in TableCurve 2D, following previously established protocols (Hiller et al., 2023). The goodness of fit r^2^ was higher than 0.984, and 90% confidence intervals were determined (dashed lines). BAC and CIP (top): The Bliss independence concentration pair at maximum synergy is not effective, located at the bottom left of the curve. For Loewe additivity the concentration pair is effective but positioned at the top right of the curve, making it not optimal (a reduction in one or both compounds may still achieve an antibacterial effect). CPC and CHX (middle): In this instance, both models resulted in not effective concentration pairs at maximum synergy. CPC and CIP (bottom): Both models resulted in effective but not optimal concentration pairs at maximum synergy.

It was also possible to determine concentration pairs at maximum synergy (*SYN_MAX*) (Table 2; Figure 1: pink outlined squares) and to calculate synergy scores (*SUM_SYN_ANT_WEIGHTED*) (Table 2) for all binary combinations against both bacterial strains with *Combenefit* (Di Veroli et al., 2016). Apart from the CPC and CIP combination against *S. aureus* and the BAC and CHX combination against *E. coli*, the concentration pairs at maximum synergy varied between the two models used and no pattern regarding their location and synergy evaluation within the concentration space of the synergy-matrices could be identified (Figure 1: synergy-matrices). Additionally, no pattern regarding the antibacterial effect could be derived for the concentration pairs as well as the individual concentrations at maximum synergy in relation to the OPECC (Figure 2). Concentration pairs at maximum synergy were more likely to be effective for Loewe additivity (Table 2: 7 out of 12 were effective, including 4 out of 6 against *E. coli* and 3 out of 6 against *S. aureus*), as compared to the pairs determined with Bliss independence (Table 2: 2 out of 12 were effective, both against *E. coli*). In both instances where the Bliss pair was effective, the corresponding Loewe pair was also effective. For the CPC and CHX combination against *S. aureus, SYN_MAX* was still negative in case of Loewe additivity. Overall, the *SYN_MAX* determined with Loewe additivity ranged from −1 to 21 and from 4 to 84 for Bliss independence (Table 2).

**Table 2:** Synergy evaluation using *Combenefit* (Di Veroli et al., 2016) for all binary combinations of BAC, CHX, CPC, and CIP against *E. coli* and *S. aureus.*

Organism	Model	Compound 1 [μg/ml]		Compound 2 [μg/ml]		*SYN_MAX*	Synergy Score	Antibacterial Effect
*E. coli*	Loewe	BAC	2	CHX	0.8	21	0.4	NE^‡^
	Bliss	BAC	2	CHX	0.8	84	11.2	NE
	Loewe	BAC	4	CIP	0.25	6	−2.2	E^§^
	Bliss	BAC	3	CIP	0.015	7	0.5	NE
	Loewe	BAC	4	CPC	1	11	0.2	E
	Bliss	BAC	2	CPC	3	54	10.7	NE
	Loewe	CHX	1.6	CIP	0.004	6	−2.6	E
	Bliss	CHX	0.8	CIP	0.015	7	−0.1	E
	Loewe	CPC	3	CHX	0.4	12	−0.2	NE
	Bliss	CPC	2	CHX	0.8	67	9.4	NE
	Loewe	CPC	4	CIP	0.5	5	−6.8	E
	Bliss	CPC	2	CIP	0.25	4	−3.1	E
*S. aureus*	Loewe	BAC	3	CHX	0.2	1	−0.9	E
	Bliss	BAC	1	CHX	0.8	52	4.1	NE
	Loewe	BAC	1	CIP	0.002	7	−6.1	NE
	Bliss	BAC	1	CIP	2.5	9	−2.6	NE
	Loewe	BAC	3	CPC	1.5	2	−0.04	E
	Bliss	BAC	1	CPC	0.75	51	3.0	NE
	Loewe	CHX	0.2	CIP	0.5	3	−13.4	NE
	Bliss	CHX	0.2	CIP	2.5	5	−3.4	NE
	Loewe	CPC	2	CHX	3	−1	−2.3	E
	Bliss	CPC	0.5	CHX	0.8	31	1.8	NE
	Loewe	CPC	0.5	CIP	0.002	11	−4.7	NE
	Bliss	CPC	0.5	CIP	0.002	12	−2.0	NE

The table presents the results calculated by the *Combenefit* software with the Bliss independence and Loewe additivity models for all binary combinations of BAC, CHX, CPC, and CIP tested against *E. coli* and *S. aureus*. For each combination pair, the table includes the *SYN_MAX metric* [defined as the “maximum synergy observed” (Di Veroli et al., 2016)], the corresponding concentration pairs at maximum synergy and their antibacterial effect, determined with the OPECC method using the curve separating effective from not effective combinations. The synergy score [*SUM_SYN_ANT_WEIGHTED metric,* calculated by *Combenefit* (Di Veroli et al., 2016)] is a quantifying measurement of synergy for the combination in general and is defined as the “sum of synergy and antagonism observed in concentration logarithmic space” (Di Veroli et al., 2016) with the additional incorporation of a „weight based on the dose response which bias the total score toward synergy achieving highest effect” (Di Veroli et al., 2016). Positive scores indicate synergy and negative scores antagonism. Ranges for no interaction vary depending on the reference model and scoring system used with, e.g., −2.0 to +2.0 being used by Khandelwal Gilman et al. (2021) for *SUM_SYN_ANT* from *Combenefit* (Di Veroli et al., 2016).

^‡^NE, not effective.

^§^E, effective.

The synergy scores determined using the Bliss independence model were consistently higher than those obtained with the Loewe additivity model, regardless of the bacterial strain or antibiotic combination tested. Positive synergy scores in both models were observed for the combinations BAC and CHX as well as BAC and CPC against *E. coli.* The synergy scores for the combinations CHX and CIP, as well as CPC and CIP, were negative for both models, regardless of the bacterial strain tested. Similarly, BAC and CIP against *S. aureus* also showed negative synergy scores for both models. For the remaining combinations, the results varied: while the Bliss independence model indicated positive synergy scores, the Loewe additivity model yielded negative scores (Table 2).

## 4 Discussion

The antiseptics CHX, BAC and CPC, which are commonly applied to disinfect skin and mucous membranes, and the antibiotic CIP were used in binary combinations in this study. To evaluate such binary combinations in the context of antimicrobial peptides (e.g., cecropin A, melittin and pexiganan), the Loewe additivity model is favored when both peptides affect the same bacterial structure, whereas the Bliss independence model is more appropriate for situations where the peptides target different structures (Yu et al., 2016). Multiple models should be used to evaluate the results from combination experiments as recommended (Chou, 2010; Di Veroli et al., 2016). With the exception of CPC and CIP against *E. coli,* the *SYN_MAX* values derived with the Bliss independence model were higher than those from the Loewe additivity model, indicating a more synergistic evaluation (Table 2). A similar pattern was observed with the synergy score (*SUM_SYN_ANT_WEIGHTED*). A positive synergy score indicates a greater effect than predicted by the respective model (Di Veroli et al., 2016; Khandelwal Gilman et al., 2021). The Bliss synergy scores were higher than those from the Loewe model (Table 2), therefore suggesting that the Bliss model tends to predict less effective outcomes compared to the Loewe model due to the differences in their calculations. This aligns with observations that Bliss independence can yield more synergistic and Loewe additivity more antagonistic evaluations (Goldoni and Johansson, 2007). Consequently, within the limitations of our study considering that we tested only six binary combinations against two bacterial strains, applying the Bliss independence model to compounds with highly similar mechanisms of action may lead to false-positive synergy evaluations, whereas using the Loewe additivity model for compounds with independent mechanisms may produce false antagonistic evaluations.

The results of synergy evaluation depend on the chosen model, which is inherently tautological since only one model can accurately describe the interaction of the compounds at a time. The two substances either behave additively (Loewe) or independently (Bliss), meaning that one model will provide a reliable prediction, but not the other. To select the appropriate model, a general understanding of the compounds’ mechanisms of action is essential. Given the differing mechanisms between CIP and the disinfectants, the Bliss independence model is likely to be a suitable approach. In contrast, the similar mechanisms of BAC and CPC, and to a lesser extent CHX with CPC or BAC, suggest that the Loewe model may be more appropriate (Baeder et al., 2015; Yu et al., 2016). Our findings reflect this distinction. For instance, the BAC and CPC combination showed synergy scores closer to 0, indicating less deviation from the model’s prediction when evaluated with the Loewe additivity model compared to the Bliss independence model (Table 2).

It remains undecided whether synergy is concentration dependent or is an inherent characteristic of a specific drug combination (Meyer et al., 2020). In our study both, synergy for individual concentration pairs (*SYN_MAX*) and the overall combination effect (*SUM_SYN_ANT_WEIGHTED*) were evaluated. Additionally, we used OPECC and its determining curve as evaluation criteria regarding antibacterial effectiveness (Hiller et al., 2023).

CIP, as an antibiotic, exhibits a markedly different mode of action compared to the other antiseptic compounds. The substances additionally differ in the time required to exert their effects, complicating direct comparisons between these types of AMS. As a result, it is difficult to determine a so-called “steady state” (Goldoni and Johansson, 2007), where the methods can be applied. This discrepancy may also influence the synergy evaluation in our study, partly due to a shorter incubation period (3 h instead of 24 h), which could be particularly relevant for the combinations containing CIP (Goldoni and Johansson, 2007).

The Loewe additivity and Bliss independence models evaluate drug interactions by comparing observed effects with predicted outcomes based on their respective assumptions — additivity for Loewe and independence for Bliss (Bliss 1939; Di Veroli et al., 2016; Loewe and Muischnek, 1926). To apply these models, an initial fit of the single dose–response curve is required, typically using the Hill equation or its variations, such as the Median-Effect equation (Chou, 2006; Di Veroli et al., 2016). Factors such as curve steepness, the choice of sigmoid or logarithmic fitting functions, and raw data processing can influence these assessments (Duarte and Vale, 2022). Since the fitting process directly impacts the results, it may introduce bias toward synergy or antagonism, influencing the precision of determined concentration pairs (Duarte and Vale, 2022; Fuentealba-Manosalva et al., 2023).

The OPECC method, however, is model-independent and distinguishes between effective and not effective concentration combinations. Originally measured two-dimensional OD-values are fitted in three-dimensional space without requiring raw data processing, thus representing the real situation. Notably, no knowledge of the interaction of the two tested substances is necessary. While the resulting fitted two-dimensional curves in the three-dimensional space may provide insight into these interactions, their interpretation is not necessary. The resulting functions of the original data are analyzed and the calculated inflection point is used as the *Optimal Effective Concentration Combination* (Hiller et al., 2023; Figure 2: red dot).

Figure 1 shows three examples of *Combenefit* synergy matrices for Bliss independence (top row) and Loewe additivity (bottom row), with the concentration pairs at maximum synergy (Table 2) for each model highlighted in pink (Figure 1). It is unlikely that the concentration pairs at maximum synergy coincide with the OPECC exactly, since the OPECC can be selected from all possible concentration combinations, whereas the concentration pair at maximum synergy is restricted to the predetermined, experimentally tested concentration pairs.

Figure 2 shows three possible outcomes for the concentration pairs at maximum synergy from the Bliss independence and Loewe additivity models (Table 2; Figure 1: pink highlighted concentration pairs) in relation to the OPECC curve (Figure 2).

1. Effective and not effective concentration pairs (e.g., BAC and CIP): The concentration pairs at maximum synergy, determined by both models, were on opposite sides of the curve that separates the effective from the not effective concentration pairs. The resulting effective concentration pair was not optimal. Although BAC and CIP tend to act independently, the concentration pair at maximum synergy derived with Bliss independence is in the not effective range (Figure 2: top).

2. Not effective concentration pairs (e.g., CPC and CHX): Both concentration pairs at maximum synergy were positioned to the bottom left of the curve and thus both were not effective. No superiority of one of these results over the other could be identified (Figure 2: middle).

3. Effective concentration pairs (e.g., CPC and CIP): Both concentration pairs at maximum synergy were situated to the top right of the curve, being effective, but not optimal. Notably, the concentration pair at maximum synergy derived with Bliss independence is lower than that of Loewe independence, which is in line with the fact that CPC and CIP tend to act independently (Figure 2: bottom).

Without additional calculations or alternative methods, it is impossible to determine the most synergistic concentration pair overall if it is not among the tested concentrations. Additionally, the effectiveness of concentration pairs is not considered in the synergy evaluation, as the synergy score, is derived from all results in the corresponding synergy matrices (e.g., Figure 1) (Di Veroli et al., 2016). The *SUM_SYN_ANT_WEIGHTED metric*, implemented in *Combenefit*, addresses this issue by providing a more comprehensive evaluation of drug combinations by assigning greater weight to concentration pairs that produce stronger effects. This approach tries to ensure, that synergy assessments are not disproportionately influenced by ineffective combinations (Di Veroli et al., 2016). Despite those added implementations, an antagonistic synergy score did not necessarily indicate that the results within the effective concentration range were also antagonistic, nor did a synergistic score always correlate with synergy in this range. Figure 2 (top) shows such an example for BAC and CIP against *E. coli,* evaluated using the Loewe additivity model. Despite a synergy score of −2.2 (Figure 1; Table 2), all tested effective concentration pairs showed either synergistic or “Loewe additive” (Tang et al., 2015) results. A similar situation was found for BAC and CHX against *S. aureus,* evaluated using the Loewe additivity model (Table 2).

The determined concentration pairs at maximum synergy exhibited inconsistent antibacterial assessments compared to the respective OPECC and its determining curve (Figure 2: three different possible outcomes). For the individual concentrations at maximum synergy, in relation to the OPECC, no pattern regarding the antibacterial effect could be determined.

Additionally, no pattern was identified for the concentration pairs at maximum synergy between the two synergy models. The effective concentration pairs at maximum synergy were not optimal, suggesting that a reduction in one or both compounds may still achieve an antibacterial effect. In contrast, the not effective concentration pairs would require higher concentrations of one or both compounds to achieve an antibacterial effect. Therefore, concentration pairs determined by Bliss independence and Loewe additivity models may require further validation and modifications before being applied against pathogens.

In such case, if the OPECC method was not applied and not effective concentration pairs have been determined as maximally synergistic, further treatment strategies would be necessary. Among others, one such strategy is the use of the LTPR (Latest Time Point of Retreatment) principle (Schramm et al., 2020). LTPR is derived from a normalized regrowth curve, enabling the assessment and comparison of not effective binary antibacterial treatments. It designates the latest time point at which retreatment should be applied to prevent bacterial regrowth (Schramm et al., 2020).

A method to evaluate synergy based on effective concentration pairs already exists in the form of the Fractional Inhibitory Concentration (FIC) index (Berenbaum, 1978). However, the thresholds for classifying interactions such as synergism, partial synergism, indifference, additivity and antagonism vary depending on the author or the guidelines followed (Berenbaum, 1978; Botelho, 2000; Dawis et al., 2003). Due to a lack of exact calculation of minimal inhibitory concentrations and effective concentration pairs, the FIC method cannot directly determine optimal concentration pairs without modification of the technique either.

OPECC meanwhile does not directly evaluate combinations. Rather, it provides a way to interpret the obtained data and prepare it for further use. The only possible recommendation regarding the general use of a combination is whether the concentration values at OPECC are lower than the effective concentrations in single applications (Table 1, *Single*) (Hiller et al., 2023). In contrast, model-dependent synergy evaluation methods can use both point-based metrics (e.g., concentration pairs at maximum synergy) and global metrics (e.g., synergy scores) to provide a general indication of whether using a combination is meaningful. However, global metrics can be influenced by data from not effective concentration ranges or concentration ranges not relevant for future application, potentially leading to false-positive or false-negative interpretation. In our experiments, the OPECC values are derived from multiple independent replicates ensuring biological relevance and statistical robustness. This allows experimental artifacts to be excluded. Achieving a similar degree of reliability for point-based synergy metrics would require similar experimental effort. The global metrics also are computed based on the point based metrics. Consequently, while global metrics can provide useful summary information, they should be interpreted alongside, rather than in place of, biologically and statistically validated point-specific data in this context.

*Combenefit* requires at least three concentrations per compound to function (Di Veroli et al., 2016), with additional concentrations improving the accuracy of predictions and, consequently, the results. Similar restrictions do not necessarily apply to OPECC: as long as an initial three-dimensional fit can be achieved from the data, OPECC can be determined, and using more data points will further improve the reliability of the final results. While model-dependent methods use only the single-application data for the initial fit (to determine dose–response curves), OPECC incorporates all measured data into the fit, thereby increasing its accuracy.

From a computational point of view, well-established model-dependent methods such as *Combenefit* or *SynergyFinder* are easier to use, as standalone software or web applications are available. In comparison, OPECC requires manual fitting of both the 3-dimensional surface and the 2-dimensional curve, making it more labor-intensive. However, unlike many model-dependent methods, this approach does not require raw data processing.

OPECC is currently limited to binary combinations, whereas model-dependent methods can also evaluate ternary and higher-order combinations. For multiple-drug analyses, however, such models must be applied with caution, as increasing the number of agents complicates both the interpretation of results as well as the classification of mechanisms of action as, in case of Bliss and Loewe, independent or additive. Notably, Bliss independence has been shown to retain accuracy when used with up to ten mechanistically different antibiotics, whereas Loewe additivity loses its predicitve power under these conditions (Russ and Kishony, 2018).

While the present study focuses on antimicrobial agents, OPECC is not inherently restricted to this domain. Similar to classical frameworks such as Loewe and Bliss, the method can, in principle, be applied to any procedure aimed the eradication of pathogens, wherever a defined target effect can be set (Hiller et al., 2023). This includes areas, such as oncology and combination pharmacotherapy in chronic disease, as well as more diverse systems such as combining antimicrobial photodynamic therapy with disinfectants (Maisch et al., 2024). Moreover, OPECC enables the determination of optimal effective concentration pairs based on conceivable optimality criteria including side effects, production costs, or compound availability (Hiller et al., 2023). Further research is needed to validate its utility across these broader pharmacological contexts.

## 5 Conclusion

In this study we compared the model-independent OPECC method with classical model-dependent synergy evaluation methods (Bliss independence, Loewe additivity). A summary of this comparison is presented in Table 3.

**Table 3:** Comparison of the model-independent OPECC method with model-dependent synergy evaluation methods (Loewe additivity, Bliss independence).

Aspect	OPECC	Loewe additivity, Bliss independence
Underlying assumptions & Mechanistic applicability	No knowledge of the interaction of the two tested substances is necessary. Applicable without prior mechanistic insight.	Requires a general understanding of the compounds’ mechanisms of action to select the appropriate model. Using an inappropriate model may lead to false-positive or false-negative synergy interpretations.
Data processing requirements	Uses directly measured OD-values. Fits all measured binary combination data in 3D space and extracts the separating curve; OPECC is defined as its inflection point.	Requires initial fitting of single-agent dose–response curves (e.g., Hill equation) and normalizes effect scale (e.g., 0–100%). Fitting choice can influence results and bias interpretations (Duarte and Vale, 2022; Fuentealba-Manosalva et al., 2023).
Output and interpretability	Identifies the *optimal effective concentration combination* (OPECC) separating effective from not effective regions. No evaluation or global metric. (Figure 2)	Provides both point-based (e.g., maximum synergy) and global metrics (e.g., synergy score), allowing general recommendations for combination use. Global metrics may be distorted by non-relevant concentration ranges, no statement on the effectiveness (neither for point-based nor global metrics) is made. (Figure 2)
Ability to predict untested combinations	Can determine points beyond tested concentration pairs through fitted curves of original data (Table 1)	Only concentration pairs tested get a synergy value assigned; global metric to evaluate combination in general (Table 2)
Experimental effort	It is reasonable to test at least 4 to 5 single concentrations; Similar experimental effort to model-dependent methods.	Requires at least three single concentrations for initial fitting, which, from a mathematical point of view, may be too few considering the far-reaching implications of this fit; Similar experimental effort to OPECC.
Computational effort	Higher manual computational workload due to individual 3D surface fitting and fitting of 2D curves. No dedicated software currently available.	Lower computational barrier; implemented in standalone software or web tools (e.g., *Combenefit, SynergyFinder*). Automated processing after initial raw data processing.
Applicability to higher-order combinations	Currently limited to binary combinations; ternary evaluation not feasible without further development.	Applicable to ternary and higher-order combinations; chosing the appropriate models gets more important considering their strong assumptions and implications
Risk of misinterpretation	Low risk from model mismatch (model-independent), but no direct synergy scoring. (Figure 2)	High if model selection mismatches the interaction type; may yield misleading synergy or antagonism results. (Figure 2)

In summary, OPECC is a model-independent method that identifies the optimal effective concentration combination where no prior knowledge of the interaction of the two tested substances is necessary. It uses directly measured data without raw data processing, fitting all binary combination measurements to determine the inflection point separating effective from not effective regions, and thereby avoids biases arising from model assumptions or irrelevant concentration ranges. In contrast, model-dependent methods such as Bliss independence and Loewe additivity rely on predefined interaction assumptions, necessitate accurate single-agent curve fitting and raw data processing, and provide both point-based and global synergy metrics. While these models can classify interactions as synergistic or antagonistic, their validity depends on appropriate model selection. Inappropriate application may result in misleading interpretations. OPECC is currently limited to binary combinations and requires more manual computational effort, whereas established model-dependent tools are automated and can handle higher-order combinations, albeit with reduced interpretability as complexity increases.

Model-dependent methods quantify interaction types via point-based (e.g., concentration pair at maximum synergy) and global metrics (e.g., synergy score) but, by design, make no statement about the antibacterial effectiveness of individual concentration pairs. As such, their results cannot be applied directly without additional calculations or experimental validation. Moreover, their validity depends on appropriate model selection and even then can lead to false-synergistic or false-antagonistic interpretations of the effective ranges.

OPECC requires no prior mechanistic insight, uses directly measured binary combination data without raw-data processing, and identifies the inflection point of the curve separating effective from not effective concentration ranges. This yields an *Optimal Effective Concentration Combination* that can be adapted to practical constraints such as side effects, cost, or availability (Hiller et al., 2023). While synergy models and OPECC address different questions, their combined use offers complementary perspectives - interaction classification on one hand, and precise, effectiveness-based dosing on the other. If the goal is to determine an optimal effective concentration pair directly from measured data, without requiring knowledge of the interaction of the two AMS tested, the calculation of the OPECC should be considered.

## Data Availability

The raw data supporting the conclusions of this article will be made available by the authors, without undue reservation.
